# Parenting perspective on the psychosocial correlates of adolescent sexual and reproductive health behavior among high school adolescents in Ethiopia

**DOI:** 10.1186/s12978-019-0734-5

**Published:** 2019-05-21

**Authors:** Belete Yimer, Wassachew Ashebir

**Affiliations:** College of Medicine and Health science, Department of Public Health, Debre Markos University, Debre Markos, Ethiopia

**Keywords:** Parenting styles, Parenting practices, Adolescence, Socioeconomic status, SRH behaviors, HIV/AIDS preventions

## Abstract

**Background:**

While parents are a crucial part of the social environment in which adolescents live, learn and earn, they could play important roles in efforts to prevent adolescent sexual and reproductive health (SRH) risk behaviors and promote healthy development. Involving parents in prevention programs to risky SRH practices in adolescents requires understanding of the effect of different parenting practices and styles on these behaviors. The purpose of this study was to investigate the relationships between various aspects of perceived parenting and self-reported engagement in sexual risk behavior among adolescents.

**Methods:**

A cross-sectional study was employed among 406 randomly selected 14–19 years old high school adolescents in Legehida district, Northeast Ethiopia from 15 February to 15 March/ 2016. Structured and pre-tested self-administered questionnaire adapted from the Youth Risk Behavior Surveillance questionnaire was used for the data collection. Bivariate and multivariate logistic regression analysis with odds ratio along with the confidence interval of 95% were used. *P*-value < 0.05 were considered for statistical significance.

**Results:**

About two-third (64.5%) of the participants reported that they had ever had sex. Nearly half (48.6%) of the participants who were currently sexually active reported that they engaged in at least one type of risky sexual behavior. Specifically, 42.7% reported starting sexual life earlier, 32.2% having more sexual partners in the past 12 months and 23.8% never used condom during the most recent sexual intercourse. High quality parent─adolescent relationships (AOR = 0.53; 95% CI (0.45–0.63) and authoritative form of parenting (AOR = 0.74; 95% CI (0.61–0.92) were associated with lower odds of engaging in risky sexual behaviors in adolescents. The odds of risky sexual behaviors were about three-fold higher in adolescents who perceived parental knowledge as poor (AOR = 2.97; 95% CI (1.51–4.25) and to some extent (AOR = 3.00; 95% CI (1.43–5.55) toward SRH than those whose parents were very knowledgeable. Adolescents with poor behavioral beliefs on SRH issues had a 37% increased odds of engaging in risky sexual behaviors.

**Conclusions:**

Therefore, to engage the parents within preventive interventions design to support healthy SRH behaviors among adolescents, the role of authoritative parenting style, and improved quality of parent-adolescent relationship, as well as improving adolescents’ behavioral beliefs and parental knowledge towards SRH are essential.

## Plain English summary

The aim of this study was to investigate the parenting correlates of sexual and reproductive health (SRH) risk behaviors in adolescents. Understanding the influences of parenting on adolescents’ sexual behaviors is important to refine parents’ roles in prevention programs to SRH risk behaviors in adolescence. Four hundred six high school adolescents in Legehida district, Northeast Ethiopia were participated in this study. Self-administered questionnaire was used to measure the main variables of socio- demographic characteristics of students and their parents; sexual behaviours of students, their parents’ parenting behaviors, quality of parent-adolescent relationship, and students’ behavioral beliefs toward SRH issues. The results suggest that SRH risk behaviors in adolescents was significantly related to parenting style and quality of parent-adolescent relationship, as well as adolescents’ behavioral beliefs and parental reproductive health knowledge. Therefore, it is essential to address these important factors within preventive interventions design to support healthy SRH behaviors among adolescents.

## Background

Adolescence is the period from about the age of eleven to the late teen years, and represents a transitional stage from childhood to adulthood [1]. An important dimension of socio-emotional development during this period is sexual and reproductive growth and development— a task that entails integrating physical, cognitive, and social domains of functioning [2, 3]. Social context influences how biological urges are channeled into behavior and adolescents’ conceptions of sexuality [4]. Though most adolescents have optimism to be resilient in absorbing setbacks and overcoming problems by representing a positive force in a society, they usually receive contradictory messages on how to address daily choices including sexuality, which have lifelong consequences for healthy development [5].

Africa accounted about 80% of the estimated five million young people living with HIV [6], and unwanted pregnancy has been inflicting about 25% of the four million unsafe abortion among the adolescents [7, 8]. About two-third of the Ethiopia’s population are young and they are the ones whose reproductive health services utilization is low and are the perpetrated with various sexual and reproductive health (SRH) problems [9, 10]. The 2011 Ethiopian Demographic and Health Survey report indicated that 0.2% of females and 0.1% of males within the age 15–19 year were infected with HIV [11]. Furthermore, about one third of pregnancies occurring during this age are unintended [12]. The common assumptions for young people acquiring sexual behaviors related health problems are, most underestimate their own risks and know little about most STIs or how to protect themselves. The prevailing potential sources of SRH information for the young people are their peers whom their knowledge are infirmed/equally ignorant or from school which is blamed for the lack of sustainable behavioral changes or from media and religious institutions that occur infrequently [13–15].

While parents and families are a crucial part of the social environment in which adolescents live, learn and earn, they could play important roles in the healthy development of adolescents [16–18]. However, the parenting challenge of adolescence is to offer opportunities for adolescents to develop and practice autonomy while providing protection from danger and the consequences of poor decisions [18]. Identifying parental variables predictive of adolescents’ behavioural outcomes has been of greatest interest to researchers. Parenting style is typically defined by level of parental warmth and control with four key parenting styles have been distinguished: authoritarian parents (strict control without being supportive); permissive/ indulgent parents (high support in the absence of strict control); neglectful/ uninvolved parents (neither supportive nor controlling); authoritative parents (high support and strict control). The research on parenting practices as they relate to adolescent behavioural outcomes has focused on several important parenting constructs including, but not limited to, parental monitoring; and parent-child communications [18].

Taken together, parenting entails a delicate balance of warmth and support, communicative relationships, monitoring, and limit-setting and enforcement in which adolescents seek and receive guidance from parents and parents provide developmentally appropriate freedom and decision-making ability [16–18]. Research suggests that this mutual reciprocity in the relationship may enhances the likelihood that adolescent will become secure, appropriately respond to stress, and be receptive to the specific parenting practices and strategies utilized to achieve advantages in distinct domains of socialization [19]. Evidences have shown that parent–child communication about SRH—specifically, open conversations characterized by warmth, support, and humor—is associated with later onset of sexual activity and reductions in sexual risk taking [20–22].

The high rates of unintended pregnancy, abortion, and STIs including HIV/AIDS make it imperative that, the development of effective SRH interventions needs to be based on knowledge about contextual influences and for adolescents, parental factors may be of particular interest. Although the government of Ethiopia has set strategies that encourage parents’ participation for better health, development and wellbeing of Ethiopian adolescents [23], little research has been dedicated to understanding the parenting context and practices whereby parents that are successful in building health-promoting behaviors do so on an every day basis. Thus, the aim of this study was to build upon and extend the current literature on parent–adolescent SRH behavior interactions by investigating the relationships between perceived parenting behaviors and self-reported engagement in sexual risk behavior among a sample of high school adolescents. Results of our study are helpful for a better understanding of the considerable influences that the dynamics of related parenting behaviors/ practices may have on promoting healthy SRH related behaviors. In addition, the findings can be employed in strategies and programmes in order for parents to promote positive SRH parenting practices on the regular basis in efforts to protect the young from engaging in risky sexual practices and associated adverse health consequences.

## Methods

### Study setting, population and design

A cross-sectional study was conducted in Legehida district, Northeast Ethiopia between 15 February and 15 March/ 2016 among high school adolescents. There are two high schools in the district, Almazbum high school is located in urban kebele (kebele is the smallest administrative unit in Ethiopia) and Shikif high school is in the rural kebele of the district. These high schools are providing educational services from grade 9 to 10. The total number of the source population was 1721 students (male, 1048 and females, 673); Shikif high school = 12 classrooms (627students) and Almazbum high school = 22 classrooms (1094 students) [24]. The study population were high school adolescents in randomly selected classrooms of grade 9 and 10 of the two schools primarily living with their parents. Parents in this study denote biological mother/father or female/ male guardians with whom the adolescents were primarily living the last twelve months prior to the study.

### Sample size and sampling procedure

A total of 409 study subjects were estimated using early sexual initiation of 41%, confidence interval of 95%; margin of error of 5% and none response rate of 10%. Two stage sampling technique was employed in order to select the required number of subjects. The number of sampled students proportional to their population size were calculated from each school and divided in to grade 9th and 10th. Half of randomly selected classrooms from each school, 11 from Almazbum (i.e., 7 classrooms from grade 9 and 4 classrooms from grade 10) and 6 from Shikif (i.e., 4 classrooms from grade 9 and 2 classrooms from grade 10) were included in the study. A sampling frame was the lists of high school students in the 17 classrooms that was obtained from students’ record office. Average number of students in each classrooms was 56. Sample classrooms and students from each classroom were selected using simple random sampling technique.

### Data collection

Structured and pre-tested self-administered questionnaire adapted from the Youth Risk Behavior Surveillance questions [25] and translated into local Amharic language was used for the data collection. The questionnaire covered items assessing socio- demographic characteristics of students and their parents, sexual behaviours of students, their parents’ parenting behaviors, quality of parent-adolescent relationship, and students’ behavioral beliefs toward SRH issues. After receiving written consent and verbal assent from the students, questionnaire was distributed and completed by the students at school. Data were collected by four thoroughly trained grade twelve completed data collectors and supervised by two primary school teachers and the principal investigators.

Questionnaire was pretested before running the actual study. The draft was tested with 20 students that were not included in the main study to check if any questions, or wordings were perceived as difficult to understand, easy to misunderstand, vague or ambiguous, strange, or irrelevant. Alternative wordings, or ways of asking questions were discussed with them to enhance the understanding and relevance of the questionnaire for the target groups. Based on results from the pretest, the draft questionnaire was slightly modified to fit our populations of interest.

### Measurements

Sexually active participants who answered agreeably to questions to any of: sexual debut up to age 15 years and/or unprotected sex and/or multiple sexual partners within the last year were classified as engaging in *risky sexual behaviors* (outcome variable). *Parent-adolescent SRH communication* was participants’ self reported having talked about sex with their parents in the past 12 months, including any of the following topics: body changes occurring during adolescence/sexual maturation, abstinence, HIV/AIDS, unintended pregnancy, condom, sexual pressure from friends/ potential partners?’ [26, 27]. Participants who responded affirmatively to the questions that ‘if their parents paying attention to know where they are after school, who they are with and what they do in their free time or when they are not at home’ were classified as having *parental monitoring.* Participants’ perceived *quality of parent-adolescent relationship* was measured based on their self reported degree of satisfaction with respect to closeness, conflict resolution, emotional support, time spent together, and communication with their parents using a 9-item parent adolescent relationship satisfaction measure [28, 29]. A sample question include ‘I am satisfied with the amount of time my parent and I spend together’. Responses were classified into high and low quality relationship using those participants who responded agreeably to at least 5 or more questions for high. Parenting style was measured using statements about parents to which adolescents indicated their perceived level of the warmth and control provided by their parents. Accordingly, *authoritarian* ‘they tell me exactly what to do’; *authoritative* ‘they ask my opinion but they have the final say’; *permissive* ‘they trust me to decide for myself’; *neglectful* ‘they do not care what I do, so I decide for myself’. An adolescent is said to have good *behavioral attitude toward sexual issues* if they agree with four and above out of six attitude questions and poor otherwise. A sample item includes ‘you think it is good to start sexual intercourse at an earlier age’. Adolescent perception on parental reproductive health knowledge classified into know very well, know to some extent or poor. Household socio economic status (SES) was rated based on a 1 to 5 scale with poorest households being rated as 1, and the wealthiest households rated as 5. The two lowest categories were combined to form a measure of low, the middle level remains as one category, and the two highest categories were collapsed to form a measure of high wealth index.

### Statistical analysis

Data were double entered and cleaned in EPI data software version 3.1. The entered data were exported and analyzed using SPSS version 21. Descriptive measures such as frequency, proportions, mean and standard deviations were computed. The associations of the independent variables entered in logistic regression with risky sexual behaviors were assessed by comparing the crude odds ratios (COR) and adjusted odds ratios (AOR) together with respective 95% CI. Only covariates that have *p*-value < 0.2 at the bivariate analysis were entered to multivariable model to control for all possible confounding factors. A *P*-value of < 0.05 was considered statistically significant.

## Results

### Socio- demographic characteristics of respondents

Of the total 409 students initially planned, 406 completed the questionnaire, making the response rate of 99%. Participants were between the ages of 14–19 years and the mean age was 16.8 (±1.09) years. The majority (72.9%) of the participants identified as Muslim religion followers. More than half (54.2%) of the participants were reported living with both of their parents. With respect to family SES, 32.2 and 60.3% living in households with low and middle income respectively (Table 1).

**Table 1:** Characteristics of adolescents in Legehida district, Northeast Ethiopia, 2016 (*n* = 406)

Variable	Category	Number	Percentage
Sex	Male	206	50.7
	Female	200	49.3
Age	14–16	368	90.6
	17–19	38	9.4
Religion	Orthodox	110	27.1
	Muslim	296	72.9
Grade level	9th	262	64.5
	10th	144	35.5
Perceived academic performance	Outstanding	227	55.9
	Average	133	32.7
	Poor	46	11.4
Living arrangement	With biological father and mother	220	54.2
	With biological father only	32	7.9
	With biological mother only	38	9.4
	With others	116	28.6
Religious service attendance	Never	68	16.7
	Occasionally	176	43.3
	Daily	162	39.9
Family residence	Rural	324	79.8
	Urban	82	20.2
Father education	Illiterate	98	24.1
	Literate	308	75.9
Mother education	Illiterate	146	36.0
	Literate	260	64.0
Family SES	Low	131	32.2
	Middle	245	60.3
	High	30	7.5

### Parental characteristics and adolescent sexual behaviours

The parental characteristics and sexual behaviours of the sample are presented in Table 2. Authoritarian parenting was reported by 53.8% of the participants. With respect to relationship satisfaction, 46.5% of participants reported a high-quality relationship with their parents. Only 11.6% of the participants reported that their parents know about SRH very well. Two hundred sixty two (64.5%) of adolescent students who participated in this study had ever had sex. The mean age at first sexual intercourse was 15 (±1.6) years. Of those who ever had sex, nearly half (48.6%) of the participants reported engaging in at least one type of sexual risk taking activities. Of those sexually active, 42.7% of respondents reported starting sexual life earlier, 32.2% having more sexual partners in the past 12 months and 23.8% never used condom during the most recent sexual intercourse. The overlap between adolescents self-reported engagement in different forms of sexual risk behaviors is shown in Table 2.

**Table 2:** Parental factors and sexual history of adolescents in Legehida, Northeast Ethiopia, 2016 (*n* = 262)

Variables	Frequency	Percentage
Parenting style		
Authoritarian	142	53.8
Neglectful	20	7.7
Permissive	32	12.4
Authoritative	68	26.1
Parent─adolescent SRH communication		
Yes	101	38.7
No	161	61.3
Parental monitoring		
Yes	143	54.7
No	119	45.3
Parent─adolescent relationship quality		
Low	140	53.5
High	122	46.5
Perception on parental SRH knowledge		
Know very well	30	11.6
Know to some extent	164	62.6
Poor	68	25.8
Adolescents behavioral beliefs of SRH issues		
Good	106	40.6
Poor	156	59.4
Adolescents’ self-reported engagement in different forms of sexual risk behaviors		
Engaged in any type of individual risk behaviours	127	48.6
Started sexual life earlier	112	42.7
Had at least two or more sexual partners in in the past 12 months	84	32.2
Never used condom during the most recent sexual intercourse	62	23.8
The overlap between adolescents’ sexual activities		
Sexual life earlier and multiple sexual partners	46	17.5
Sexual life earlier and never used condom during the last sex	25	9.5
Multiple sexual partners and never used condom during the last sex	2	0.6
All forms	29	11.2

Table 3 showed the associations of the parental environment with risky sexual behaviors among adolescents. Unadjusted analysis showed that risky sexual behavior was associated with perceived parents as authoritative (COR = 0.65, 95%CI; 0.58–0.89), communicating SRH issues with parents (COR = 0.49, 95%CI; 0.25, 0.99), parents with poor SRH knowledge (COR = 3.17, 95%CI; 1.65, 4.37) and know SRH to some extent (COR = 1.35, 95%CI; 1.14, 6.23) as well as reporting high quality relationship with parents (COR = 0.73, 95%CI; 0.32, 0.95). As for adolescents’ self-reported of their behavioral beliefs, poor SRH beliefs was associated with self-reported engagement in risky sexual activities (COR = 1.85, 95%CI; 1.11, 2.48).

**Table 3:** Associations of parental factors with sexual risk behaviours among Legehida high school adolescents, Northeast Ethiopia, 2016 (*n* = 262)

Variables	Sexual risk behaviours		COR (95% CI)	†AOR (95%CI)
	Yes	No		
Parent─adolescent relationship quality				
High	54 (44.3%)	68 (55.7%)	0.73 (0.32, 0.95)	0.53 (0.45, 0.63)*
Low	73 (52.1%)	67 (47.9%)	1.00	1.00
Parenting style				
Authoritarian	54 (38.0%)	88 (62.0%)	1.00	1.00
Neglectful	14 (70.0%)	6 (30.0%)	0.26 (0.09, 1.19)	0.44 (0.27, 1.32)
Permissive	25 (78.1%)	7 (21.9%)	0.17 (0.01, 1.04)	0.32 (0.19, 1.18)
Authoritative	34 (50.0%)	34 (50.0%)	0.65 (0.58–0.89)	0.74 (0.61–0.92)*
Parental monitoring				
Yes	63 (44.1%)	80 (55.9%)	0.68 (0.33, 1.46)	0.81 (0.51, 1.28)
No	64 (53.8%)	55 (46.2%)	1.00	1.00
Parent─adolescent SRH communication				
Yes	38 (37.6%)	63 (62.4%)	0.49 (0.25, 0.99)	0.73 (0.50, 1.07)
No	89 (55.3%)	72 (44.7%)	1.00	1.00
Perception on parental SRH knowledge				
Poor	44 (64.7%)	24 (35.3%)	3.17 (1.65, 4.37)	2.97 (1.51, 4.25)*
Know to some extent	72 (43.9%)	92 (56.1%)	1.35 (1.14, 6.23)	3.00 (1.43, 5.55)*
Know very well	11 (36.7%)	19 (63.3%)	1.00	1.00
Adolescents behavioral beliefs of SRH issues				
Good	61 (57.5%)	45 (42.5%)	1.00	1.00
Poor	66 (42.3%)	90 (57.7%)	1.85 (1.11, 2.48)	1.37 (1.79, 2.18)*

Key: *COR* Crude odds ratio, *AOR* Adjusted odds ratio, *CI* confidence interval; †Adjusted for all variables in the table and additionally for the socio demographic variables; *p-value < 0.05

In the multivariate analysis, participants reported their perception of parental SRH knowledge, parenting style, quality of their relationship with parents and own behavioral beliefs of SRH issues were independently associated with sexual risk behaviors among adolescents adjusting for all variables in the table and additionally for socio-demograpic variables. Accordingly, high parent─adolescent interactions quality (AOR = 0.53, 95% CI; 0.45–0.63) and authoritative parenting (AOR = 0.74, 95% CI; 0.61–0.92) were associated with lower odds of engaging in risky sexual behaviors in adolescents. The odds of risky sexual behavior was three-fold higher in adolescents whose parents had poor (AOR = 2.97, 95% CI; 1.51–4.25) and some (AOR = 3.00, 95% CI; 1.43–5.55) SRH knowledge than those their parents know SRH very well. Adolescents with poor behavioral beliefs about sexuality had a 37% increased odds of engaging in risky sexual behaviors.

## Discussion

The study showed the prevalence of having experienced sexual risk behaviors was at 48.6%. The higher sexual risk behaviors were predicted with poor SRH attitudes and perceived parental knowledge as poor and to some extent toward SRH while authoritative parenting, and high adolescent-parent relationships quality were associated with reduced sexual risk behaviors.

In this study, authoritative parenting was associated with lower odds of engaging in risky sexual behaviors. Previous studies also showed a protective effect of authoritative parents against risky sexual behavior [30–33]. The finding might be explained by the evidence that authoritative parents’ use of open discussion, joint decision-making, and firm but fair limit-setting helps adolescents feel valued, respected, and encouraged to think for themselves [34, 35]. In addition, while adolescents with authoritative parents have been found to report higher overall life satisfaction [36], it is also possible that an authoritative parenting style may be associated with increased satisfaction with their relationship with their parents, which may be associated with healthier SRH behaviors.

Our measure of parent-adolescent relationship quality assessed adolescent satisfaction with a range of aspects of the relationship, including closeness, conflict resolution, emotional support, time spent together, and communication found that high quality parent-adolescent relationship was associated with reduced SRH risk behaviors of adolescents studied. This is in line with previous studies conducted in other part of Ethiopia [30] and elsewhere [37, 38] in which parent child connectedness was strong predictor. This may imply that high quality relationship can create an easy environment for parents to organize and plan regularly shared family activities, such as television co-viewing and discussion, shared dinners, or parental monitoring that have been shown associated with lower rates of sexual activity [32, 33].

Our results are consistent with previous study [32], which found that adolescents from parents with poor and to some extent SRH knowledge were more likely to engage in risky sexual behaviors. The possible explanations is, on the one hand, that despite the strong value parents place on SRH education, it is also possible they do not have the basic knowledge and skills of SRH to draw upon to help foster their adolescents’ healthy SRH behaviors on a day-to-day basis. On the other hand, studies have shown that the level of parental knowledge is largely correlated with the presence of SRH communication [39–42]. Their results showed that, adolescents who considered their parents’ knowledge of SRH as poor were less likely to experience SRH communication with them, which, in turn, may limit their access to information regarding SRH through their parents and increase their odds of sexual risk behaviors. This implies that there is a need to parental intervention strategies and programs designed to equip parents at least with the basic knowledge and skills of SRH which might help them translate their strong values towards SRH behaviors into everyday parenting practices.

Present study observes that adolescents with poor behavioral beliefs about sexuality had increased odds of engaging in risky sexual behaviors. This might go with a theoretical ground that an individual’s attitudes and intentions significantly influence his or her actions [43]. Communicative and self-disclosing supportive relationships with parents is recommended to improve adolescents’ views of normative sexual behavior that potentially influenced by their peers, media and neighborhood [20–22]. This is supported by the finding of reduced SRH risk behaviors among adolescents having high quality relationship with their parents. While communicating SRH issues with parents was not independently associated with sexual risk behavior in adolescents, it did show association in the bivariate logistic regression analysis, indicating the risk of sexual risk behavior was 51% lower in respondents who reported communicating than in those who did not. This is of interest. Such results emphasize the importance of parent-adolescent relationship quality and communication as key interventions of adolescent SRH.

Unlike previous studies [30, 32, 33], the present study did not confirm association between parental monitoring and sexual risk behaviors. This finding should be interpreted with caution as the present study could be underpowered to observe such associations. The results could also be an indication of difference in the relative importance of exposures in different settings due to contextual variations.

This study might have had some limitations that should be considered when interpreting our results. First, all measures were collected by self-report and such data can be affected by information bias of different kinds which could explain the observed absence of association between parental monitoring and sexual risk behaviors. Second, it dealt only with the adolescents’ perspective, which might be different from what their parents might really have perceived, so assessing the issue from both perspectives would provide a broader picture. Third, the fact that the study was limited to high school adolescents who were found at school during the time of data collection was another limitation as it may be difficult to generalize the results to out of school that might have different life experiences. Despite these limitations, this study takes a valuable step toward identifying adolescents who are at sexual risk behaviors and outcomes, as well as avenues for key interventions.

## Conclusion

Nearly half of the participants who ever had sex were engaged in at least one type of sexual risk behavior. The odds of sexual risk behaviors was higher among adolescents with poor SRH attitudes and perceived parental knowledge as poor and to some extent toward SRH. However, authoritative parenting, and high adolescent-parent relationships quality reduce sexual risk behaviors among adolescents in this setting. Therefore, our findings suggest that the need to address family variables focusing on the most positive form of parenting aspect or the role of authoritative parenting, and improved quality of parent-adolescent relationship within preventive interventions design to support healthy SRH behaviors among adolescents. Additionally, improving adolescents’ behavioral beliefs and norms towards SRH and improving parental education to help parents promote healthy SRH behaviors making positive modifications (e.g., parent-adolescent relationship quality and communication) into everyday parenting styles/practices are essential in order to reduce SRH risk behaviors and associated outcomes among adolescents.

## Abbreviations

AOR: Adjusted odds ratio
CI: Confidence interval
COR: Crude odds ratio
HIV/AIDS: Human immunodeficiency virus/Acquired immunodeficiency syndrome
SES: Socio economic status
SRH: Sexual and reproductive health
